# Physiology and pathophysiology of mucus and mucolytic use in critically ill patients

**DOI:** 10.1186/s13054-025-05286-x

**Published:** 2025-02-07

**Authors:** Thomas Roe, Thomas Talbot, Isis Terrington, Jayant Johal, Ivan Kemp, Kordo Saeed, Elizabeth Webb, Rebecca Cusack, Michael P. W. Grocott, Ahilanandan Dushianthan

**Affiliations:** 1 General Intensive Care Unit, University Hospital Southampton NHS Foundation Trust, Tremona Road, Southampton, SO16 6YD UK; 2 Perioperative and Critical Care Theme, NIHR Southampton Biomedical Research Centre, University Hospital Southampton/University of Southampton, Southampton, SO16 6YD UK; 3 Integrative Physiology and Critical Illness Group, Clinical and Experimental Sciences, Faculty of Medicine, University of Southampton, Southampton, SO16 6YD UK

**Keywords:** Mucolytics, Mechanical ventilation, Intensive care, Critical care

## Abstract

Airway mucus is a highly specialised secretory fluid which functions as a physical and immunological barrier to pathogens whilst lubricating the airways and humifying atmospheric air. Dysfunction is common during critical illness and is characterised by changes in production rate, chemical composition, physical properties, and inflammatory phenotype. Mucociliary clearance, which is determined in part by mucus characteristics and in part by ciliary function, is also dysfunctional in critical illness via disease related and iatrogenic mechanisms. The consequences of mucus dysfunction are potentially devastating, contributing to prolonged ventilator dependency, increased risk of secondary pneumonia, and worsened lung injury. Mucolytic therapies are designed to decrease viscosity, improve expectoration/suctioning, and thereby promote mucus removal. Mucolytics, including hypertonic saline, dornase alfa/rhDNase, nebulised heparin, carbocisteine/N-Acetyl cysteine, are commonly used in critically ill patients. This review summarises the physiology and pathophysiology of mucus and the existing evidence for the use of mucolytics in critically ill patients and speculates on journey to individualised mucolytic therapy.

## Introduction

Mucus was described the by Hippocrates as one of the four humours of the body. It is a highly specialised fluid secreted on the apical surfaces of epithelial tissues that acts as an interface between the individual and their environment [1, 2]. Mucus consists of water, proteins, lipids, carbohydrates, and electrolytes and provides a physical and immunological barrier to pathogens [3, 4]. Moreover, it acts as a lubricant, aids air humidification, and provides a selectively permeable barrier for gas exchange and nutritional absorption [5, 6].

Mucus dysfunction in the airways is common in primary chronic respiratory conditions such as asthma [7], chronic obstructive pulmonary disease (COPD) [8], cystic fibrosis (CF) [9], and lung cancer [10]. However, in acute intensive care unit (ICU) settings, where patients require mechanical respiratory support, a multitude of factors including infection, accumulation of inflammatory cells, airway dehydration, reduced cough reflexes, supra-normal oxygenation, and mechanical stress from ventilation can lead to changes in mucus characteristics and increased mechanical impedance to mucociliary clearance. This, in turn, causes mucus accumulation, airway plugging, and subsequent worsening in oxygenation, ventilation, and infection [11–15].

Managing secretions in mechanically ventilated patients is crucial for effective weaning and prevention of secondary respiratory tract infections [16]. Mucolytic agents, in general, aim to reduce viscosity and improve airway mucus clearance. A survey conducted in the United Kingdom (UK) in 2020 suggested that 83% of ICUs use mucolytic agents within normal practice, primarily for “thick secretions” [17]. Surprisingly, only 4% of centres follow guidelines to inform their mucolytic prescribing decisions, which might be explained by the limited evidence guiding their use in critical care [18–20].

In this review, we aim to provide a comprehensive overview of the physiology and pathophysiology of mucus in critical illness. We further explore the scientific rationale and the evidence for the use of different mucolytic agents in critically ill patients. Specifically, we aim to highlight some key factors and complexities of mucolytic choice in this clinical context and explore the possibilities offered by individualised mucolytic therapy.

## Physiology of respiratory mucus

The respiratory epithelial lining shows progressive morphological changes from nose to alveoli with ciliated epithelium and to more simple cuboidal epithelium distally [21]. Secretory and ciliated cells contribute to the production and movement of mucus. While [21]tracheal secretory cells found within submucosal glands expel secretions via ciliated ducts, secretory cells in the smaller airways are found on the epithelial lining secreting directly into the airway lumen [22, 23].

Mucus producing cells contribute around 60% of total gland volume [24], producing isotonic mucus of up to 98% water (variable depending on the clinical context), salts (1%), and mucin glycoproteins (0.3%), which has antimicrobial, immunological and defensive properties [2, 25–27]. Mucins are complex glycoproteins that create the dense gel layer of mucus. They are rich in cysteines at their terminal ends which bond monomers together. Mucin monomers, although few, are extremely large molecules (> 200 k Da) [28]. After polymerisation, the meshwork possesses a high avidity for water, allowing mucus to act as a non-Newtonian liquid [29]. Central regions of mucin monomers contain tandem repeats of proline, serine, and threonine allowing O-linked glycosylation [1]. Glycans attach to these regions producing complex compounds with diverse functions. Recent studies indicate that glycan configuration may directly ameliorate microbial pathogenic virulence [4, 30–33]. Disruption of glycosylation or glycan structure is also associated with disease and critical illness [4, 34].

Mucins are encoded by 17 MUC genes [35, 36] and once transcribed, they are translated and stored as granules in the goblet cells before being secreted. Initially in a dehydrated state and once in the respiratory tract, they rapidly hydrate causing swelling and expansion due to their size, sugar content, and polymerisation into long chains [37–39]. In the human respiratory tract, MUC5AC and MUC5B predominate. MUC5B is constantly expressed, whereas MUC5AC can be induced by pathogens, gases, inflammatory mediators, adrenergic, cholinergic, and neurohumoral factors [36, 40–43]. Mucins can also be membrane bound and these (namely MUC1, MUC4, MUC16, and MUC20) are present on epithelial cells in the respiratory tract [44] and involved in immunological processes [28, 45, 46]. The preferred model of mucus within the airways is the “gel on brush” model. Mucus closest to the epithelial cells forms the periciliary layer (the “brush”) where the cilia interact with a thinner liquid regulating mucus movement. Above this, a thicker “gel” layer captures pathogenic organisms which are then destroyed by host immune cells [46, 47].

On the epithelial surface, the Cystic Fibrosis Transmembrane Conductance Regulator (CFTR), calcium activated chloride channels, and sodium channels regulate the water content within the periciliary and gel layer via osmosis. The depth of the periciliary layer is maintained by adenine and uridine nucleotides. In in-vivo models of normal ventilation, cyclical pressure alterations on human bronchial epithelial cells induced calcium dependent ATP and MUC5AC secretion in the airway epithelium [48]. These nucleotides, in response to mechanical stress in the airways activate P2Y2 and A2b receptors on ciliated cells, altering the sodium and chloride composition of the periciliary layer, leading to increased water movement into the lumen which facilitates mucus movement [28, 45, 49, 50]. The brush layer prohibits dehydration by the mucus gel layer and limits the passage of noxious particles > 40 nm into the brush layer, and > 5 nm at the apical border of the epithelial cell [47].

Mucus is transported from the lower respiratory tract to the pharynx, where it is expelled or swallowed, a process known as mucociliary clearance [51]. Ciliated cells, found as distally as the terminal bronchioles, drive this mucus movement. The cilia comprised of motor proteins beat 12–15 times per second moving the mucus about 1 mm per minute [52–54]. These ciliated cells are stimulated mechanically and by irritants [55]. While the exact mechanism of mucus transport remains poorly understood, there are different models describing both a continuous (blanket) layer or a discontinuous (globular/flaky) movement [56, 57].

## Pathophysiology of mucus in critical illness

Mucus dysfunction in critical illness can arise from both the primary disease and the treatments administered in ICU (Table 1). Most studies extrapolate pathophysiological processes from animal models, but in-vivo studies aiming to determine true pathological changes in human disease are lacking [58].

**Table 1:** Summary of the alterations in mucus and respiratory epithelium during critical illness

Characteristic	Changes in critical illness
Epithelium	Hyperplasia and metaplasia of mucus secreting cells [59–61]
	Epithelial to mesenchymal transformation [62]
Mucociliary transport	Reversal of flow direction, regardless of positive pressure ventilation [61, 63]
	Slower transit time [61, 63]
	Thicker proportion of periciliary “brush” layer [64]
	Reduction in cilia length [65]
Immune response	Activation of dendritic cells [64]
	Induction of cellular adhesion molecules in respiratory capillaries [62, 66]
	Induction of fibrosis [62]
Mucus composition	Increased mucus viscosity [67]
	Increased DNA and proteinaceous components within mucus [68]
	Induction of MUC5AC, MUC5B, MUC1 [59, 60, 62, 69, 70]
	Increased cleavage of MUC4 and MUC16 [59, 60, 62, 69, 70]

Mechanical ventilation, whilst necessary in critical care, increases the risks of lung trauma, ventilator associated pneumonia, and physical/respiratory deconditioning leading to adverse clinical outcomes [71]. Few studies have explored mucus changes during invasive mechanical ventilation. Koeppen et al., demonstrated a 58-fold increase in MUC5AC glycoprotein in bronchoalveolar lavage samples of intubated patients with acute lung injury [68]. Using in-vitro models of human tissue, they demonstrated that mechanical stretch could induce a 2.4-fold increase of the MUC5AC gene via Nuclear Factor Kappa B (NFkB). Other studies also indicate pro-inflammatory mediators induce MUC5AC via NFkB [66]. In murine models of invasive mechanical ventilation simulating high mechanical stress, MUC5AC deletion was associated with increased survival, reduced neutrophil dysfunction, and dampened neutrophil production of inflammatory cytokines compared to wild type mice with the same ventilatory exposure [68].

Furthermore, in acute respiratory distress syndrome (ARDS) secondary to SARS-CoV-2 infection, several disease specific mechanisms exist to induce mucus dysfunction. Adivitiya et al., described on interactome including 32 protein interactions and clusters of mucus pathogenesis [72]. One such cluster describes the SARS-CoV-2 virus’s ability to bind to the Angiotensin receptor, causing NFkB upregulation and subsequent mucin transcription, translation, and mucus secretion to supra normal levels [72, 73].

Powell et al., compared subglottic aspirates of patients who had been mechanically ventilated for a median of five days to newly intubated patients, identifying significantly higher levels of MUC5B (but not MUC5AC) [74]. The subglottic mucin from ventilated ICU patients caused impaired neutrophil function with a reduced ability to neutralise *P. aeruginosa* (commonly seen in ventilator associated pneumonia) [74]. This implies that the variations in the type of mucin may influence local immune response and development of secondary bacterial infections in ventilator associated pneumonia.

While the effects of smoking, age, and chronic respiratory diseases on mucociliary function are well established [75, 76], mechanisms in acute illness are less well studied. In mechanically ventilated patients, impaired mucociliary clearance is implicated in mucus dysfunction. It is hypothesised that positive pressure ventilation causes mucus to flow towards the lower airways with the overall movement following the bias flow during ventilation (either inspiratory or expiratory) [61, 63]. Nakagawa et al., compared the mucociliary clearance using saccharine transit time (STT) between non-intubated ICU patients and healthy volunteers. They demonstrated that the STT was progressively impaired even without mechanical ventilation in critically ill patients [67]. This suggests critical illness regardless of mechanical ventilation can be associated with ciliary dysfunction.

The deleterious effects of hyperoxia in critical illness are becoming clearer with ongoing studies comparing more conservative to standard higher oxygen targets [77]. A recent study in neonatal murine models found that hyperoxia reduces cilia length, decreases motor proteins, and causes mitochondrial dysfunction in ciliated cells [65]. These findings are consistent with human postmortem studies [78]. Both murine and canine models of invasive mechanical ventilation in critical illness show reduced mucociliary clearance in hyperoxic conditions, although mechanistic data is limited [79, 80].

Prolonged mechanical ventilation is also associated with cellular changes including goblet cell hyper/metaplasia, increased expression of membrane bound MUC1, and increased cleavage of MUC4 and MUC16 leading to increased production of pro-inflammatory mediators [59, 60, 62, 69, 70]. While such changes are intended to provide innate immune response to combat respiratory pathogens, in a prolonged critically ill state, there is expression of altered cell surface receptors (including vimentin, N-cadherin) that are associated impaired ventilation, worsening lung compliance, and fibrous tissue deposition [62]. In addition, murine models of lung injury with invasive mechanical ventilation demonstrate epithelial mesenchymal transition and early indicators of fibrotic changes after only four hours of mechanical ventilation [62]. In summary, critical illness and mechanical ventilation are associated with significant alterations in mucus physiology that may lead to adverse clinical outcomes. However, in vivo studies investigating these in human models are limited.

## Mucus sample collection and analytical techniques

The development of diagnostic, prognostic, and therapeutic targets based on mucus analysis has the potential to improve outcomes of those experiencing mucociliary dysfunction during critical illness. Collection and analysis of mucus is crucial for understanding the impact of critical illness and ICU therapies on mucociliary pathophysiology in mechanically ventilated patients. While mucus obstruction has been well studied in chronic conditions such as CF and COPD [81], these may not be applicable to critically ill patients with acute respiratory conditions. With the advancement of analytical strategies, point of care mucus testing and individually tailored therapeutic strategies may be possible [82]. For now, despite the many sampling and analysis methodologies, technical challenges and limitations prevent routine clinical use.

Mucus sampling techniques are broadly divided into in vitro and in (or ex) vivo techniques. Use of in-vitro models utilising bronchial epithelial cells have been successfully cultured, allowing secretion, sampling and analysis of mucin in different experimental conditions [83]. While robust in vitro models likely aid further research, care must be taken to limit uncontrolled stimulation from the exogenous triggers [83]. Moreover, such techniques cannot fully replicate the human biome and are less helpful for individual patient analysis in a clinical context. In contrast, in vivo or ex vivo sampling allows direct assessment of mucus in a clinical setting and can be obtained through coughing, via the endotracheal tube or endoscopically [84]. Mucus sampling techniques, including their limitations, are summarised in Table 2 [84].

**Table 2:** Summary of mucus collection techniques

Method	Procedure details	Advantages/Disadvantages
Induced/ Spontaneous sputum	Alert patients either spontaneously cough sputum for analysis, or inhale nebulised hypertonic saline over several minutes to loosen secretions which stimulates coughing	Spontaneous collection in alert patients can provide samples from upper and lower airways with no specialist training but can be easily contaminated by saliva
		Induced samples use hypertonic saline to facilitate expectoration, however this can alter mucus composition and can be uncomfortable for patients
Endotracheal sampling	In intubated patients only, a suction catheter is inserted into the trachea/main bronchus before suction is applied and samples are collected	Sampling in intubated patients only which requires both specialist skills and equipment
		Once intubated, sample technique requires minimal technical skill required to perform
		Upper airway mucus only, with intra/inter individual variability in content and volume
Bronchoscopy/ BAL	Using a bronchoscope, the clinician enters the branchial tree and subsegments of the lungs. Using saline flushes and suctioning, samples are collected	Patients must be sedated/intubated to perform this technique, requiring both specialist personnel and equipment
		Provides therapeutic benefit in patient with mucus plugging
		Can be performed regularly for longitudinal analysis of distal airway mucus

While sputum may be obtained from spontaneously ventilating patients, this is often impractical in critically ill patients. Sputum sampling may be spontaneous or induced. Spontaneous sputum sampling requires patient effort, and induced sputum is obtained with the use of nebulised hypertonic saline to aid expectoration [85]. Both spontaneous and induced sputum risks contamination from saliva and epithelial cells, although sample quality based on overall cell viability has been shown to be improved in induced samples [86]. Moreover, the physical property of sampled sputum is highly variable and can be influenced by the method of obtaining the sputum [86]. Endotracheal sampling may be achieved in different ways. Whilst endoscopic subglottic aspiration has been used for upper airway mucus sampling in some studies [74], it is possible to obtain subglottic samples directly using endotracheal tubes that incorporate subglottic aspiration ports. Sampling the more proximal airways using endotracheal suctioning is straightforward in intubated patients [87]. While bronchoscopy is not routinely recommended [87, 88], flexible bronchoscopes are increasingly used on ICU for airway clearance and sampling of respiratory secretions, where less invasive methods are insufficient [88]. Different methods for mucus sampling may be achieved using bronchoscopy. Bronchoscopy may be used to retrieve mucus or mucus plugs from airways [82]. Bronchoalveolar lavage using instilled saline can be used to help gather less contaminated mucus from smaller airways [82]. The bronchoscope may also be used to gather endobronchial brushings and biopsies. The cells may be either directly analysed or used to develop cell cultures for ex vivo modelling and evaluation [89]. However, bronchoscopy is an invasive procedure and is not without risk. Moreover, sample quality and composition may be altered by instilled lignocaine/saline [90, 91], and the physical stress during bronchoscopy can impact neurally mediated mucus secretion [92].

**Fig. 1:**
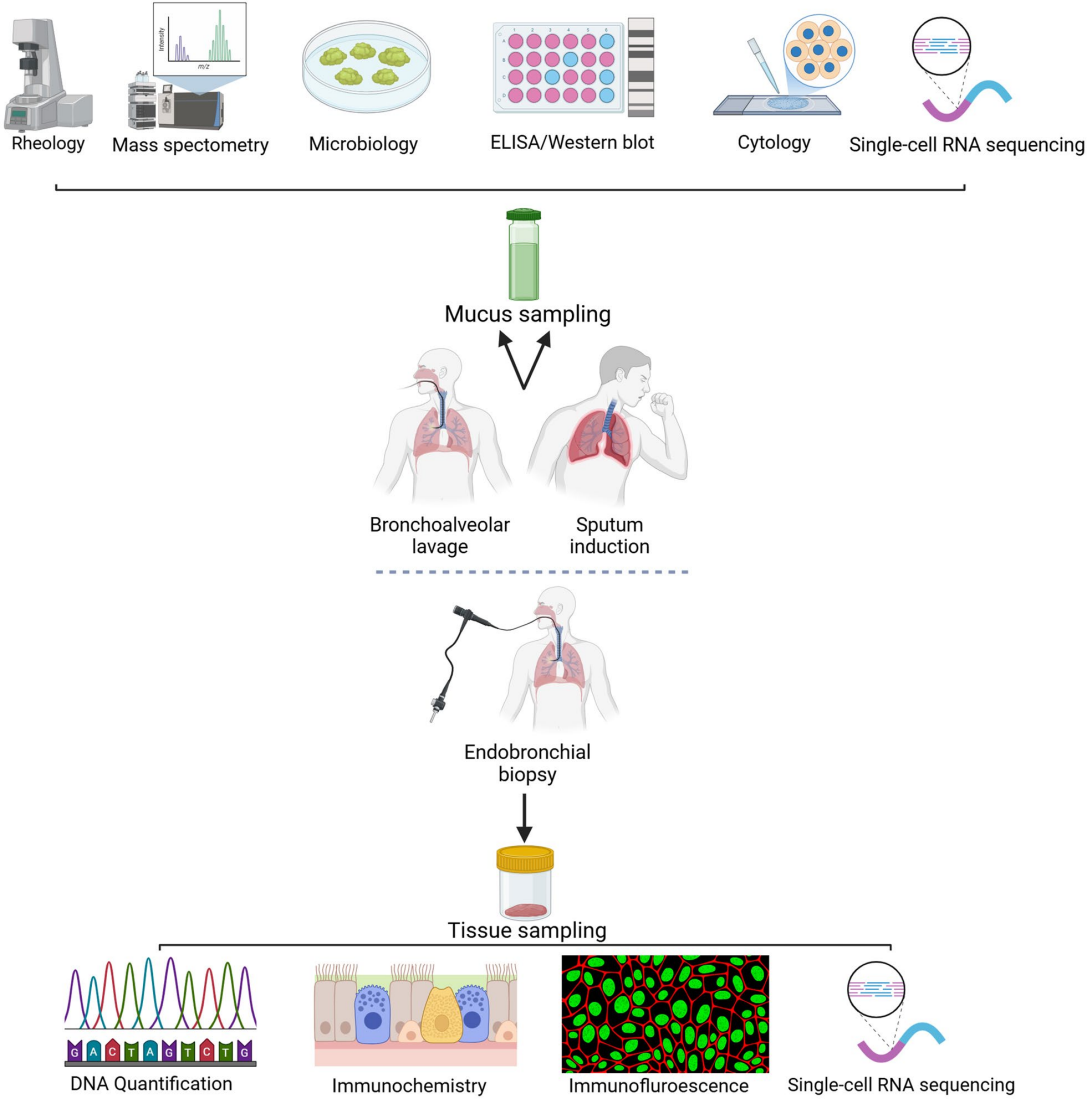
Mucus and tissue sampling with link to biochemical, microbiological, cytological, histological, rheological, genetic and proteomic analysis

Following sample collection, processing is required due to the viscus nature of mucus [85]. The fluidifying agent dithiothreitol (DTT) has been shown to improve sputum characteristics in readiness for further analysis [93], although mechanical dissolution is also possible [94]. Many analytical methods are available and some of those most relevant to clinical practice are summarised in Fig. 1 [82]. Analyses may be performed at macroscopic, cellular, or molecular levels and biophysical properties of mucus can also be evaluated. Macroscopic approaches include qualitative description of mucus and measurement of mucus solids concentration [95]. Some macroscopic analyses may be performed without mucus collection, including assessment of mucociliary transport using visualisation techniques (particularly scintigraphy) [96] and in vivo detection of mucus based on x-ray imaging [97].

Cellular analysis includes cytology, and microbiology is commonly used to help understand the cellular environment of the mucus. Cell counting facilitates removal of samples heavily contaminated with epithelial squamous cells, and characterisation of the differential cell count [85]. The quantification of neutrophils, eosinophils, macrophages and other cell lines enables phenotyping of lung inflammation [85]. The cell count can also provide baseline data when considering molecular indices (such as neutrophil count when examining concentrations of extracellular DNA and neutrophil extracellular traps (NETs)) [98]. The full scope of microbiological mucus analysis is beyond the scope of this review, but includes microscopy, microbe identification using staining and other methods, and culturing [82]. Molecular assays (such as nucleic acid amplification tests) are also increasingly used for identification of pathogens, including viruses, with increased sensitivity and specificity [99].

At the molecular level, mucus analysis involves studying of gene expression and protein content which mostly focus on identification of mucin mRNA expression within mucus samples. In situ hybridisation using labelled nucleic acid strands has been used for identification of mucins, with quantification achieved using polymerase chain reaction techniques [100]. Newer genomic techniques are being introduced, including single-cell RNA-sequencing, which has been used to study the differentiation of airway secretory cells allowing dynamic assessment of the airway epithelium [101].

Protein assessments by enzyme-linked immunosorbent assay (ELISA) may help to identify mucins directly and having greater utility with mucus samples collected in vivo [74, 84]. Various ELISA techniques have been developed, each with pitfalls and advantages. Sandwich ELISA overcomes some difficulties associated with immobilisation of mucus samples to study plates and increases sensitivity and specificity. However, it is a more complex process to undertake [102]. Mucins may also be detected using Western blotting [103]. Mass spectrometry can be used to accurately identify and quantify target proteins [43]. It can also be used to study large numbers of proteins and their interactions (including mucins and surrounding globular proteins) [104].

Protein–protein (including mucins and other globular proteins) interactions influence the physical and biological properties and its role in innate immune defence [104]. Rheometers can be employed to measure changes in flow properties caused by these interactions [104]. Physical behaviours and flow properties of mucus varies during respiratory conditions and can impact mucus clearance [29]. Rheological measurements of mucus are complex but can generally be considered in terms of measured resistance to flow (which may be described as the loss or viscous modulus or Gʺ) and tendency to recover shape following application of force (also known as the storage or elastic modulus or Gʹ) [29]. Such measurements may be performed for ‘bulk’ mucus samples and termed macrorheology [29]. For analysis of smaller areas within the mucus, micro- or nanorheological measurements are more appropriate [29]. Whilst macrorheology will consider the averaged physical properties of a larger mucus sample, micro and nanorheology can be used to describe the physical properties of smaller microenvironments within the mucus [29]. Macrorheometers such as the cone and plate rheometer make allowance for the heterogenous nature of mucus but tend to require large sample sizes [29]. Other devices include the capillary viscometer and the filancemeter (which measures how well mucus can be drawn into threads) [29, 82]. Microrheometry using magnetic devices allows for processing smaller samples, although it is considered less precise [29]. Alternatively, particle tracking motion (PTM), based on analysing the movement of beads in the sample, is a more accurate technique that uses smaller sample sizes [29]. A similar method of measuring viscoelasticity, but incorporating fluorescence labelled dextran, is used in fluorescence recovery after photobleaching (FRAP) [105].

Rheometric analysis in chronic lung disease is an expanding area of study. During acute exacerbations, it has been shown that mucus from patients with cystic fibrosis demonstrates increases in elastic and viscous moduli, but these measurements return to baseline once the exacerbation has been treated [106]. These changes in rheology are thought to be due to the increased levels of extracellular DNA and filamentous actin at times of exacerbation [106]. The role of neutrophils and increased levels of extracellular DNA in airways disease has been demonstrated in asthmatic patients [98]. Intracellular material (including DNA and cytoplasmic proteins) is released by activated neutrophils and leads to development of NETs [107]. Whilst NET formation assists neutrophils with immune defence, production can also contribute to asthma pathogenesis, in addition to many other lung diseases [107]. Human mucus rheology has been examined following exposure to NETs from stimulated neutrophils [108]. The presence of NETs has been shown to increase elastic and viscous moduli of mucus, and additionally significantly reduce the movement of nanomolecules through the mucus (measured using particle tracking methods) [108]. Rheology therefore forms a bridge between a known pathological mechanism involving NET formation and a marker of respiratory disease exacerbation and progression. It has the potential to develop the understanding of muco-obstructive conditions, including determining therapeutic targets and assessing response to therapy. It also has diagnostic potential both for chronic and acute lung disease.

Furthermore, it has been suggested that rheological assessment of the airway surface layer (ASL) in mechanically ventilated patients may allow for more targeted ventilator settings to reduce ventilator associated lung injury [109]. For now, rheology, as with the other analytical techniques described, has numerous challenges and limitations. It does, however, seem to demonstrate promise for clinical application in the future.

## The impact of humidification on mucus

Controlling humidification of inhaled gas is a vital component of the mucociliary transport system. Without optimised humidification, airway epithelium and mucosal layers can become dried leading to impaired mucociliary transportation, increased airway resistance and heightened risk of opportunistic infection [110]. For those requiring supplemental oxygen, particularly prolonged mechanical ventilation, it is critical that ventilatory gases are warmed and humidified to reduce these secondary consequences, along with optimising patient comfort. However, a careful balance is required as excess humidity can overwhelm mucociliary transportation increasing mucosal burden and causing atelectasis from water droplet formation [111].

The American Association for Respiratory Care recommends humidification for all mechanically ventilated patients. This can take place through active humidification using heated water reservoirs or passively through heat and moisture exchangers (HME) inserted into the ventilation circuit [112]. The main difference between active and passive humidification is the requirement of external power or water supply. Active humidification, such as that of bubble, pass over or counter flow humidifiers, utilise evaporation of water chambers through active heating. These add water vapour to respiratory gases independent of patient physiology. In contrast, passive humidification with HME (consisting of an encased hygroscopic membrane) inserted into the ventilation circuit, utilises the membrane properties to capture water vapour during expiration to reintroduce it during inspiration [113].

Although cheap and easily introduced into a ventilator circuit, HMEs can be problematic in ICU settings with limitations associated with minute volumes (> 10L/min), temperature control (< 32 ^°^C), higher risk of air leakage and secretion blockage compared to active humidification strategies [111]. Due to these limitations, active humidification is traditionally used in ICU, especially in those requiring prolonged mechanical ventilation or with thick/large secretion burdens [111]. Control of air temperature is also an important factor when considering humidification requirements. Active systems have temperature monitoring to limit excessive condensation and potential thermal injury to respiratory mucosa [113]. Passive systems do not require this safety feature as heating and cooling occurs secondary to physiological airflow [110].

Current BTS guidelines do not support the standard use of continuous humidification for low flow oxygen therapy (mask/nasal cannula) or during non-invasive ventilation therapy [114, 115].

## Mucolytics

Mucolytic choice within the ICU environment is subject to local guidelines and clinician familiarity, often without consideration of evidence of proven efficacy. Within this section, different mucolytic choices commonly seen in UK ICUs will be described including mechanisms of action and published evidence supporting their use. The type and cost of common mucolytic therapies used in the ICUs are detailed in Table 3.

**Table 3:** Standard Costings of commonly used mucolytic therapies available (correct as of March 2024)

Product	Dose	Cost for 7-day course (£)
Acetylcysteine 200 mg/ml IV Injection	160 mg QDS	45.32
Carbocisteine 375 mg Caps	750 mg TDS	0.81
Carbocisteine 250/5 ml Soln	750 mg TDS	10.12
Carbocisteine 750 mg Soln	750 mg TDS	3.34
Heparin 25,000units/ml Injection	25,000 units BD	99.15
NaCl 7% Nebuliser Soln	4 mL QDS	6.14
Dornase Alfa 2.5 mg Nebuliser Soln	2.5 mg BD	278.00
Ambroxol Tablet/Soln	30 mg TDS	9.10

## Hypertonic saline

**Fig. 2:**
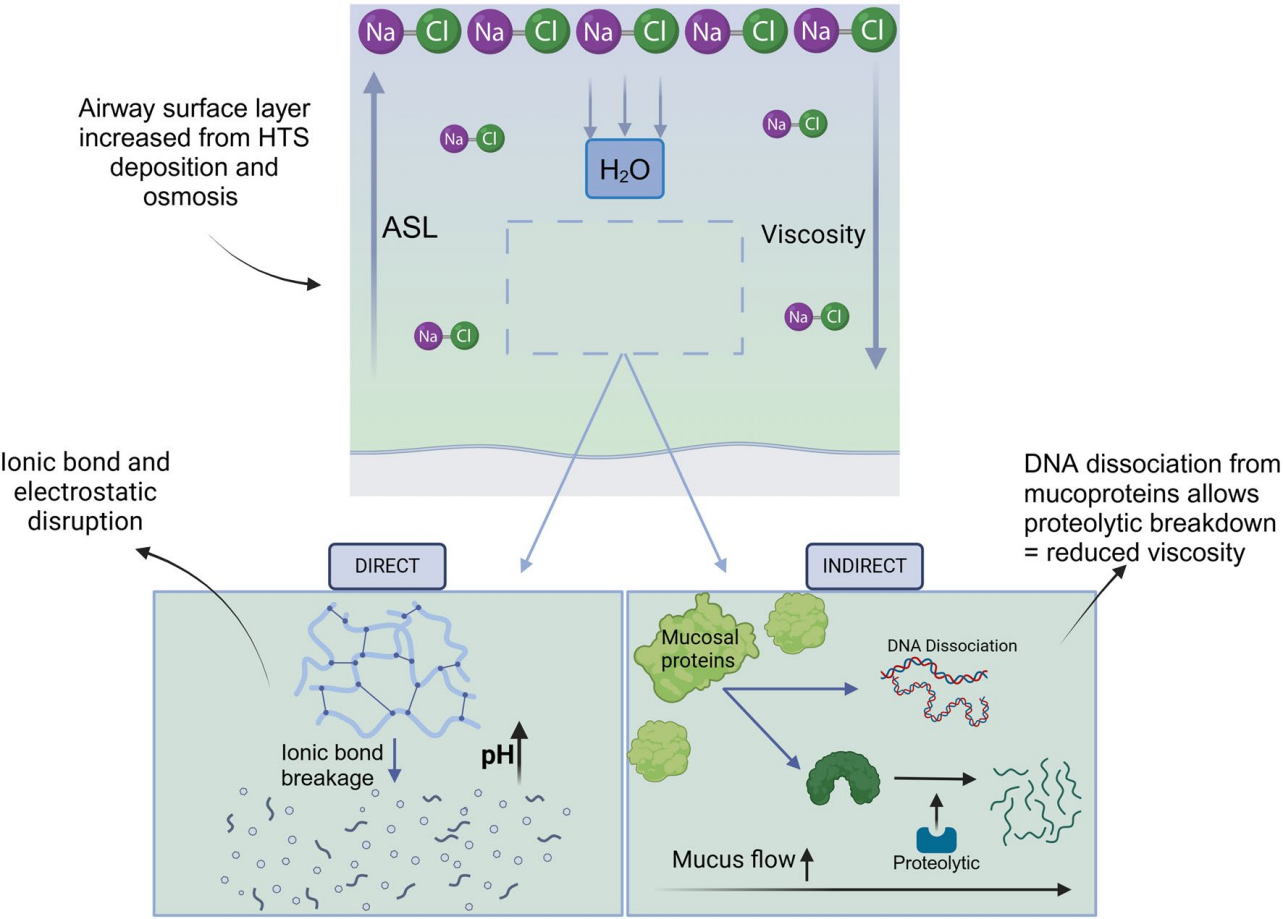
The direct and indirect actions of HTS. Direct: Deposition of itself onto the airway surface layer creating an osmotic effect leading to increased ASL height and reduced viscosity by disruption of ionic bonds in mucin-electrostatic interactions. Indirect: Repair of mucociliary mechanisms (secondary to reduced viscosity and increased water content of mucus), and dissociation of DNA from mucoproteins allowing for natural proteolytic enzymes to act

Hypertonic saline (HTS) has an osmotic pressure greater than physiologic saline (0.9% NaCl) with 3–7% NaCl typically used [116]. Though its multiple mechanisms of action make classification difficult it is typically categorised as an expectorant (Fig. 2). Direct actions of HTS involve its disruption of ionic bonds within the mucus with a reduction of cross linking and protein entanglement, and reduced viscosity [117, 118]. In-vitro HTS increases the ASL height by depositing itself onto the surface and drawing in additional water through osmosis (reducing mucus viscosity) and increasing secretory volumes. Restorations of depleted ASL can improve mucociliary function [119, 120]. Hypertonic saline nebulisation commonly triggers coughing, which also can aid mucus clearance [121]. HTS may also impact mucus through the dissociation of DNA from mucoproteins, stimulating natural proteolytic enzymes leading to reduced mucus density, which have direct protective mechanisms against oxidative injury in the ASL and may protect against microbial biofilm formations [122–124].

Hypertonic saline has a good safety profile in adults with no significant adverse effects reported. It can cause temporary irritation to the airway triggering acute coughing, hoarseness and bronchoconstriction. For this reason, it may be used in caution in patients with bronchospastic tendencies. To reduce these side effect risks, often clinicians will administer an inhaled bronchodilator before nebuliser treatment is initiated [125]. There is currently limited evidence to support the use of hypertonic saline in children.

Most clinical studies assessing HTS efficacy focus on chronic diseases, particularly CF where it has been shown to accelerate mucus discharge, improves airflow and lung function [119, 126]. However, in a 12-week crossover trial with 48 paediatric CF patients, 7% HTS was inferior to DNase in improving FEV1 [127]. When similar comparisons were undertaken in-vivo 3% HTS was preferable to 7% [128]. Due to the risk of bronchospasm at higher HTS concentrations, caution is advised in its use in patients with COPD or asthma and should be preceded by a bronchodilator [129].

Results from studies of HTS use in acutely unwell/ventilated patients have produced contradictory results. In a retrospective analysis, Altunhan et al. found that 7% HTS improved atelectasis by 70% in mechanically ventilated neonates, compared to 81% with DNase and 95% when both HTS and DNase were administered concomitantly [130]. In mechanically ventilated adult patients with acute lobar atelectasis, chest x-ray atelectasis score improved more with DNase than HTS, but without any differences in extubation rate or complications [131]. Evidence for use of HTS in the critical care setting is limited, further trials are required to demonstrate its benefit in critically ill patients.

### N-acetyl cysteine (NAC)/Carbocisteine

**Fig. 3:**
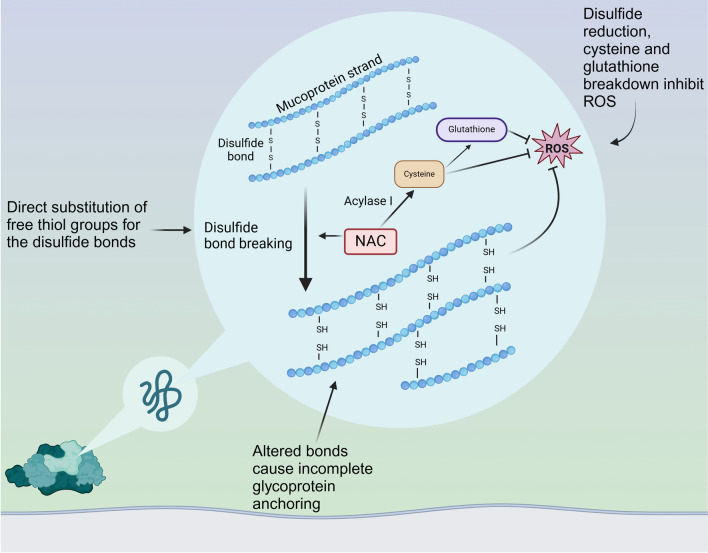
Mechanism of action of N-acetylcysteine (NAC). Breakage of the disulphide bonds causes incomplete glycoprotein anchoring and reduction in mucus viscosity. NAC acts on ROS via glutathione and cysteine breakdown but also secondary to disulfide reduction. Together these reduce viscosity and elasticity of mucus

NAC and carbocisteine are classical mucolytics and mucoregulators respectively, with distinct mechanisms of action. Erdosteine, an effective mucolytic licensed for acute bronchitis in the UK is not recommended by National Institute for Health and Care Excellence NICE thus not commonly used in the National Health Service (NHS) and is not discussed further. NAC disrupts disulphide bonds within the mucin polymer by substituting free thiol (sulfhydryl) groups for disulfide bonds, altering the 3-D structure and thus reducing viscosity and elasticity (Fig. 3) [132, 133]. NAC can be given orally, nebulised or intravenously, with nebulised being the preferred route for rapid local mucosal action [134]. Although oral NAC has yet to demonstrate any mucolytic properties [135, 136], it is used for other indications. NAC also serves as a precursor to reduced glutathione providing antioxidant benefits as a free oxygen radical scavenger [133].

The most common side effect for nebulised NAC is nausea associated with the unpleasant smell and sticky residue that may form. This residue can be easily removed with water [137]. However, a serious side effect is hypersensitivity to NAC with approximately 18% experiencing an anaphylactoid reaction leading to acute bronchospasm, angioedema and hypotension [138]. Given the significance of this reaction, a trial nebuliser should be given with close supervision before considering regular administration. Any patient who has had a previous reaction to NAC (in any medicinal form) should not be given the nebulised solution. Caution is recommended in patients with a history of peptic ulcer disease due to the risk of gastrointestinal bleeding and irritation.

Current evidence supporting the use of nebulised NAC is weak and studies have shown no significant improvements in mucous expectoration, viscosity, oxygenation, or mortality. [20, 139–142]. A systematic review and meta-analysis of 13 randomised controlled trials and 1712 patients on mucolytic agents for acute respiratory failure which included three NAC trials (two for IV use and one for nebulised) found no improvements in duration of mechanical ventilation, length of hospital stays or ventilator free days [20]. Masoompour et al., randomised 40 patients who had been ventilated for more than three days to either nebulised NAC or normal saline and found a temporary modest rise in oxygen saturation within 12 h in the NAC group [143]. Similar findings are demonstrated within the SARS-CoV-2 cohort, with a meta-analysis of four studies (355 participants) found no observable differences in intubation rate, oxygenation index, length of ICU stay, length of hospital stay, or mortality; albeit with significant variability in dose and administration route, generating an overall low certainty of evidence [144]. Overall, studies have failed to show any significant clinical improvements, and a large multi-centre randomized trial is needed to clarify the effects NAC on robust clinical outcomes [139, 143].

Carbocisteine is administered orally and acts as a mucoregulator to restore equilibrium between sialomucins and focomucins (glycoproteins that regulate viscoelastic properties of bronchial mucus) via the intracellular stimulation of sialyl transferase enzyme [145]. It may also reduce cellular damage by activating protein kinase B phosphorylation and the suppression of NF-KB and ERK1/2 MAPK signalling pathways providing antioxidant and anti-inflammatory effects [146]. Colombo et al. in 1994 using rabbit models found that carbocisteine increases chloride transport across airway epithelium which may also enhance mucociliary action [147]. Carbocisteine is recommended by NICE as a long term daily mucolytic for patients with COPD. Chronic smoke exposure causes excess mucus and oxidative stress making strategies to reduce this oxidative burden vital for effective therapy [148]. While a recent systemic review of COPD patients showed that daily carbocisteine reduced the frequency of acute exacerbations and improve quality of life scores when compared to placebo [148, 149], intensive care studies of patients with mechanical ventilation are lacking [150].

Studies have suggested potential benefits through the inhibition of bacterial adherence to ciliated epithelial cells, reducing levels of IL-6/8 and scavenging reactive oxygen species (ROS) [146, 151, 152]. Moreover, they could also improve the clinical efficacy of antibacterial agents [153]. An ongoing study of interest (MARCH trial) is examining the effect of carbocisteine on mucus hydration and clearance in intensive care/ventilated patients [154, 155]. In the meantime, more robust evidence is needed to support the use of NAC/carbocisteine as an acute mucolytic in mechanically ventilated critically ill patients.

### Heparin

Heparin is an endogenous glycosaminoglycan and part of a group of proteoglycans (including heparan sulphate, chondroitin sulphate, keratan sulphate and hyaluronic acid) commonly found within the extracellular matrices, basement membranes and endothelial cell surfaces [156]. Heparin is derived from the highly negatively charged heparan sulphate, which is found on the surface of most cell types, particularly those of vascular endothelial cells [157]. Unlike heparan sulphate, heparin is produced solely by mast cells and then stored in granules, where it makes up to 30% of the dry weight of these granules [158, 159].

**Fig. 4:**
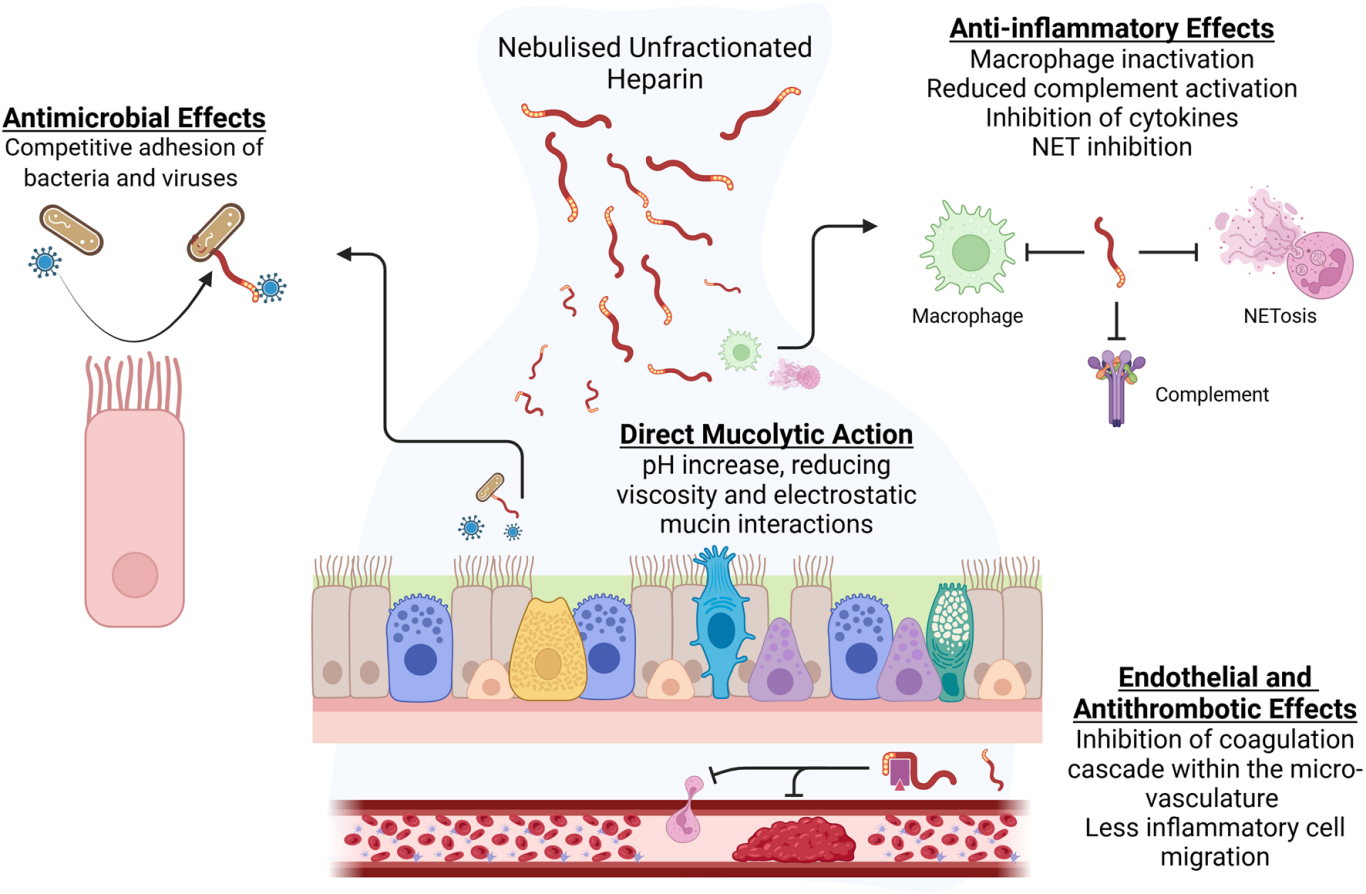
Mechanism of action of heparin as a mucolytic is multimodal. Antimicrobial effects via competitive adhesion of viruses and bacteria to epithelial wall by attaching to GAG molecules on heparin molecule. The anti-inflammatory effects produced via inhibition of inflammatory cytokines and complement cascade, and subsequent neutrophil and macrophage inhibition. Heparin acts directly in the mucus by increasing the pH, and thereby reducing viscosity and mucin interactions. The antithrombotic mechanism of heparin acts in the local microvasculature, preventing coagulation and thrombosis

The anticoagulant properties of heparin are well documented. However, several other properties including inhibition of cell growth, modulation of proteases, ability to reduce inflammation and bronchoconstriction have all been previously evaluated [149, 160–163]. The mechanism of action of heparin as a mucolytic is depicted in Fig. 4. Unfractionated heparin can be nebulised either through a face mask (via Venturi nebuliser system) or directly into endotracheal tubes (using a vibrating mesh membrane). The most common preparations are 5,000 IU in 1 ml, 10,000 IU in 2mls or 25,000 IU in 5mls [164]. Although early studies in the 1960s and 1990s reported subjective improvement in asthma and bronchoconstriction symptoms, high quality objective trial evidence of clinical benefit in the critically ill is limited [165].

Although few adverse events have been reported, there are risks associated with the administration of nebulised unfractionated heparin. Contraindications to its administration include any patient with an active or recent history of bleeding (particularly pulmonary) or any patient with a history of heparin-induced-thrombocytopenia. The incidence of blood-stained secretions and bleeding after administration is similar to that of controls [166, 167]. Although bloodstaining of secretions may occur, clinically significant bleeding is rare.

In a double-blinded, randomised controlled study of 50 mechanically ventilated patients nebulised heparin did not improve oxygenation but did show a significant increase in ventilator-free days [167]. A multi-centre trial involving 256 patients at risk of developing ARDS assessed the physical function among survivors as the primary outcome and showed there was no significant differences between patients treated with nebulised heparin and controls. However, they did observe fewer ARDS cases in the heparin group (15%) vs control patients (30%) and an improvement in 60-day survival at home with heparin (87%) vs controls (73%) [168]. Importantly, heparin was not associated with increased adverse events. Within a meta-analysis of mechanically ventilated adult patients treated with nebulised heparin there was a reduced ICU length of stay demonstrated across six RCTs, and reduced duration of mechanical ventilation found across two RCTs. However, such benefit did not extend to length of hospitalisation or mortality [169].

Within the COVID-19 cohort, the efficacy of nebulised heparin is apparent given that the diffuse alveolar damage that is induced by the SARS-CoV-2 virus is associated with significant coagulopathy and microvascular thrombosis which is attenuated by heparin [170]. Furthermore, nebulised heparin has been shown to reduce attachment and invasion of mammalian of many viruses, including SARS-associated coronavirus [171]. A recent systematic review indicated that nebulised unfractionated heparin could improve oxygenation parameters without any increase in adverse effects, however the quality of evidence is low and extrapolated from two RCTs, two observational studies, and a case report [172].

A meta-analysis of 23 studies found improvements in respiratory function (FEV1) in patients with COPD and asthma exacerbation, suggesting potential benefit of nebulised heparin in addition to standard therapy in this chronic respiratory group. Interestingly, a subgroup analysis demonstrated that unfractionated heparin proved more effective than low-molecular-weight heparin [173]. While recent evidence indicates that nebulised heparin may be beneficial, large randomised controlled trials are needed to evaluate the prophylactic and therapeutic effects in critically ill mechanically ventilated patients [169].

### Recombinant human deoxyribonuclease (rhDNase)/Dornase alfa

**Fig. 5:**
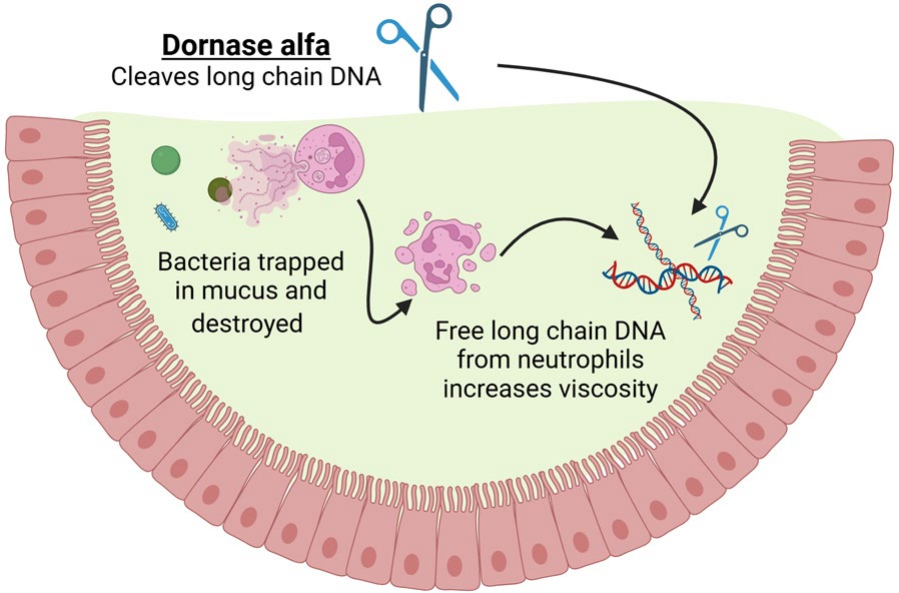
Mechanism of action of Dornase Alfa. The enzyme cleaves extracellular DNA without any effect on intracellular DNA reducing viscosity of purulent sputum. Activity is altered by the presence of calcium and magnesium. Neutrophil activation and NETosis allows successful antimicrobial clearance, however, with prolonged exposure cellular apoptosis causes free long chain DNA to enter the mucus lining. This causes increased mucus viscosity and subsequent worsening of mucociliary clearance. Dornase alfa cleaves long chain DNA fragments, reducing this effect

The lysis of inflammatory cells, particularly neutrophils release highly polymerised deoxyribonucleic acid (DNA), which contributes to increased mucus viscosity and adhesiveness (Fig. 5). Neutrophils release a meshwork of chromatin fibres, antimicrobial peptides and enzymes (neutrophil elastase, cathepsin G, myeloperoxidase) that form NETs [174]. While NETs provide defence against invading pathogens, they may also act as sources of immune effector and proinflammatory molecules that may promote tissue damage and autoimmunity. The resultant increase in viscosity can contribute to mechanical plugging of airways and increases the risk or spread of infection further [175].

Recombinant Human Deoxyribonuclease (rhDNase/Dornase alfa) reduces mucus viscosity by depolymerising extracellular DNA, reducing its size and transforming viscous mucus into a liquid [176, 177]. Dornase alfa is available as a solution administered by jet nebulisers through a face mask or directly into an endotracheal tube. The standard adult dose is 2500 IU (equal to 2.5 mg) once daily and is currently licensed only for patients with CF [178].

Dornase alfa is generally well tolerated with very few contraindications. Common side effects associated with include sore throat, hoarse voice, conjunctivitis and fever [179]. Pharyngitis is the most common with up to 40% of patients treated for 24 weeks reporting the reaction. https://www.pulmozyme.com/hcp/side-effects/summary.html. At present, current UK guidelines do not support the use of dornase alfa in children under 5 and is only licensed for CF usage. Considering these limitations and significant cost per vial, its application is limited to approved prescribers only [180].

Most literature of dornase alfa is focused on CF, with few studies conducted on other conditions such as asthma and ARDS. Recently interest in dornase alfa has grown for the treatment SARS-CoV-2. A recent small case series of 5 with SARS-CoV-2 ARDS was unable to conclude on the efficacy in secretion reduction, but it was well tolerated [181]. Holliday et al. conducted a non-randomised pilot trial of dornase alfa in ARDS patients secondary to SARS-CoV-2 and showed a significant improvement in oxygenation from baseline at day two in the intervention group without any increase in adverse events. However, this effect was not sustained following stopping of the intervention [182]. Another study of 39 patients with ARDS secondary to SARS-CoV-2 treated with an average of nine doses of dornase alfa showed a reduction in respiratory support requirements [183]. However, randomised, placebo-controlled trials are required to further evaluate intervention[184].

### Ambroxol

**Fig. 6:**
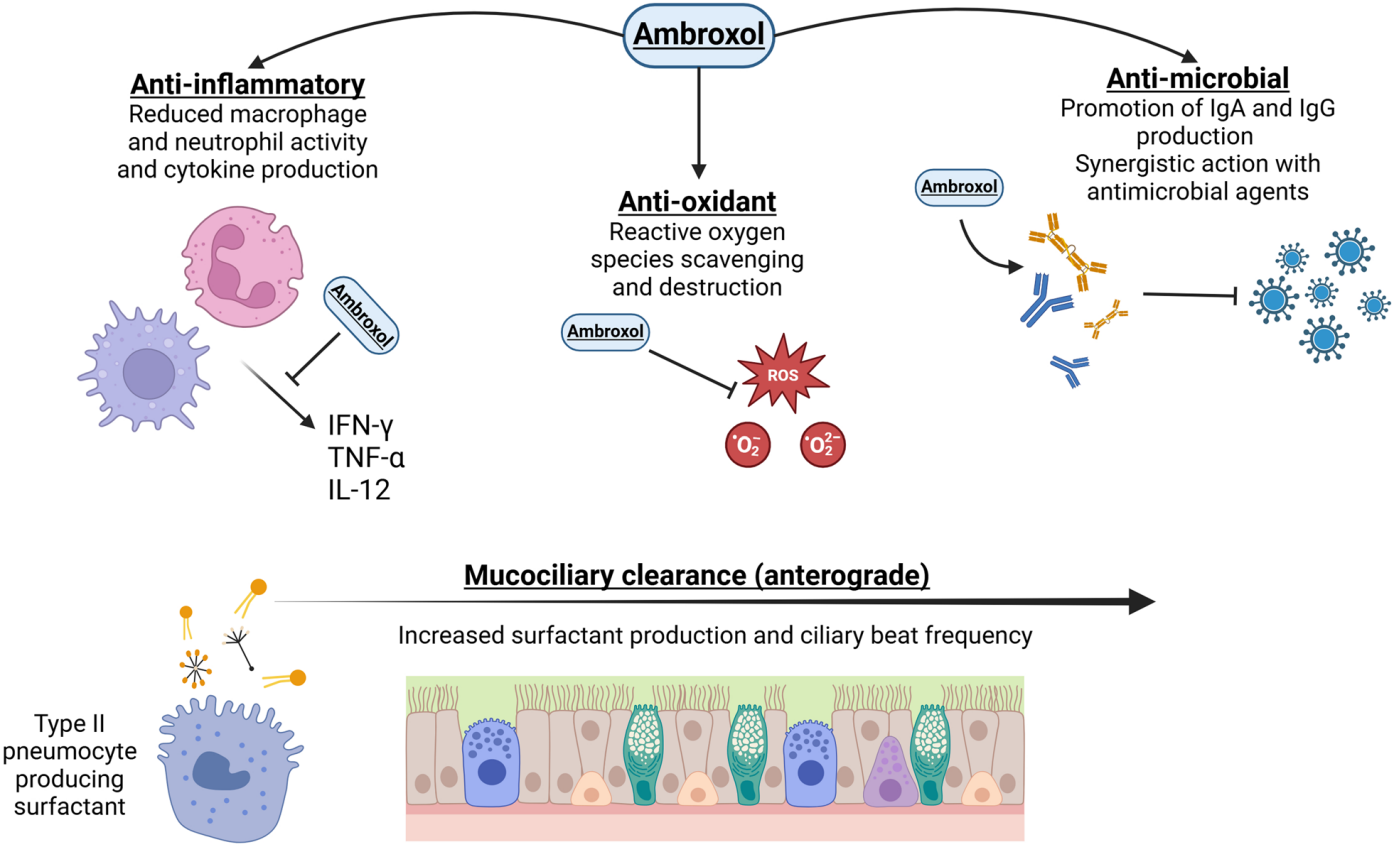
Mechanism of action of ambroxol. It possesses anti-inflammatory effects by reducing the production of pro-inflammatory cytokines. In addition, it acts as an antioxidant through reactive oxygen species scavenging and destruction. Antimicrobial effects via increased IgG and IgA secretion, and it increases stimulation of lamellar bodies within type II pneumocytes causing increased production of surfactant. Finally, ambroxol increases the ciliary beat frequency, improving mucociliary clearance

Ambroxol (2- amino-3,5-dibromo-N-[trans-4-hydroxycyclohexyl] benzylamine) has both mucolytic and secretagogue properties alongside antioxidant, anti-inflammatory, and antimicrobial properties (Fig. 6) [185]. It stimulates surfactant synthesis in type II pneumocytes. After administration, ambroxol is stored within the type II pneumocyte lamellar bodies and lysosomes, inducing structural changes to surfactant molecules, and subsequent surfactant secretion from the cell [185, 186]. Although the exact mechanism is not fully understood, effect of ambroxol may be indirect, given that in rat models, labelled phosphatidylcholine secretion (surfactant) was not stimulated by ambroxol across a variety of doses [187].

Ambroxol is reported as having minimal side effects with mild gastrointestinal discomfort being the most frequently described [188]. Caution is advised in patients with a history of peptic ulcer disease, especially if used concomitantly with other products known to irritate GI mucous membranes. Although easily sourced online and throughout Europe, ambroxol is not currently licensed within the UK and as such is not available for clinical prescriptions.

Ambroxol increases ciliary beat frequency in animal models [189]. The antimicrobial and anti-inflammatory properties of ambroxol may enhance mucus clearance. In murine models, ambroxol reduced viral multiplication by increasing IgA, IgG, and mucus protease inhibitors, whilst limiting the expression of pro-inflammatory molecules including TNF-α, IFN-γ, and IL-12 within the acute/subacute stage of Influenza A upper respiratory tract infection [190]. It also demonstrates anti-biofilm properties [191], and inhibition of endotoxin-induced lipid peroxidation [192, 193]. Human studies have shown ambroxol may also increase antibiotic levels within the lung tissue (including amoxicillin, ampicillin, and erythromycin) [194, 195].

Malerba et al. highlighted that there are > 100 studies of different design (including randomised trials), evaluating over 15,000 patients, for the clinical use of ambroxol [196]. Studies have demonstrated efficacy in a variety of adult and paediatric acute and chronic respiratory diseases [196], however evidence for use in intensive care is limited. In lung cancer patients undergoing lobectomy and patients undergoing spinal stabilisation, ambroxol reduced post-operative complications [197]. A meta-analysis of 10 randomised controlled trials including 508 patients with acute lung injury or ARDS demonstrated high does ambroxol (≥ 15 mg/kg or 1000 mg/day) for 7 days improved oxygenation with a reduction in the duration of mechanical ventilation compared to usual therapy [198].

### Bronchodilators

Bronchodilators, including salbutamol, ipratropium, and phosphodiesterase inhibitors are classically utilised in obstructive airway conditions such as asthma and COPD [199, 200]. The mechanism of action of these medications within this context are well described. These agents, particularly beta-adrenergic agonists, do however possess some mucokinetic properties, causing increased ciliary beat frequency and subsequent mucociliary clearance, although the evidence to support this is limited for use in human models of critical illness [201–204].

The bronchodilators that promote mucociliary clearance through increased ciliary beat frequency necessitate functioning cilia [205], which as mentioned, can be damaged during critical illness and the consequences of organ support within ICU environments. Furthermore, O’Riordan et al., (2022) demonstrated in a placebo-controlled trial that aerosolised salbutamol use does not result in significantly altered volume or composition of airway secretions in 14 intubated-ventilated participants [206]. Therefore, outside of chronic obstructive respiratory pathologies in acute illness, bronchodilators should be only considered for their intended purpose.

## Future considerations

**Fig. 7:**
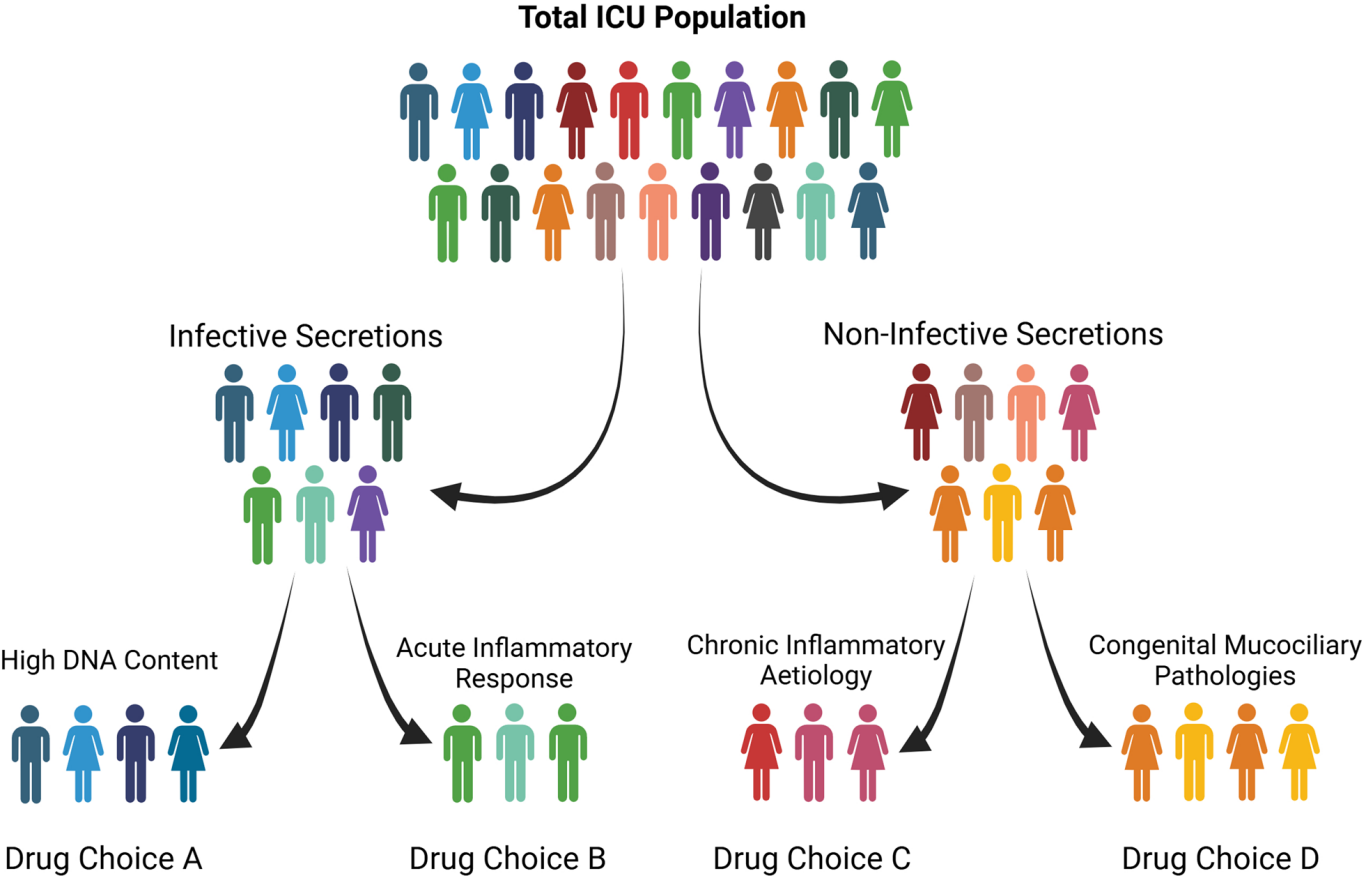
Possible phenotypes of mucus composition analysis at the bedside, embedded with multi-omics data and known patient factors to facilitate mucolytic choice

The future of mucolytic therapy in standard intensive care practice is driving towards personalised care. At present, the choice of mucolytic agents is based upon macroscopic examination of mucus consistency and colour, clinician experience, patient tolerance, local guidelines, and formulary restrictions. The mucolytic that works best for an individual patient is unclear and the heterogeneity of critically ill patients is vast. Moreover, subjective mucus analysis has been demonstrated to not correlate with measured rheological characteristics of mucus in critically unwell intubated patients [207]. Ongoing and future research will be limited by the heterogenous nature of the study population and the lack of pre-phenotyping of mucus characteristics to pre-define an ideal mucolytic regimen. While clinical trials are underway including the Mucoactives in Acute Respiratory failure: Carbocisteine and Hypertonic saline (MARCH) trial which is comparing the efficacy of carbocisteine and/or hypertonic versus standard care in critically unwell adults with a primary outcome of ventilator dependency time [154, 155], mechanistic exploration is necessary to identify the correct mucolytic for the correct patient given recent availability of rapid rheometers and development of multi-omics data analysis supported by artificial intelligence, future research should be aiming to individualise of the prescription and administration of currently used mucolytics matching them to mucus phenotypes (Fig. 7).

## Conclusion

Mechanical ventilation and critical illness are associated with significant alterations in mucus physiology. While there are several mucolytic therapies are available and commonly used, the evidence for their use in critical care setting is limited with handful of relatively low-quality studies to inform clinical practice in ICU. Detailed mechanistic studies are required to identify mucus specific characteristics to target personalised therapy and improve clinical outcomes.

## Data Availability

Not applicable in this review.
